# Selective Cleaning Enhances Machine Learning Accuracy for Drug Repurposing: Multiscale Discovery of MDM2 Inhibitors

**DOI:** 10.3390/molecules30142992

**Published:** 2025-07-16

**Authors:** Mohammad Firdaus Akmal, Ming Wah Wong

**Affiliations:** Department of Chemistry, Faculty of Science, National University of Singapore, 3 Science Drive 3, Singapore 117543, Singapore; firdaus.akmal@u.nus.edu

**Keywords:** MDM2, drug repurposing, selective cleaning, machine learning, docking

## Abstract

Cancer remains one of the most formidable challenges to human health; hence, developing effective treatments is critical for saving lives. An important strategy involves reactivating tumor suppressor genes, particularly p53, by targeting their negative regulator MDM2, which is essential in promoting cell cycle arrest and apoptosis. Leveraging a drug repurposing approach, we screened over 24,000 clinically tested molecules to identify new MDM2 inhibitors. A key innovation of this work is the development and application of a selective cleaning algorithm that systematically filters assay data to mitigate noise and inconsistencies inherent in large-scale bioactivity datasets. This approach significantly improved the predictive accuracy of our machine learning model for pIC_50_ values, reducing RMSE by 21.6% and achieving state-of-the-art performance (R^2^ = 0.87)—a substantial improvement over standard data preprocessing pipelines. The optimized model was integrated with structure-based virtual screening via molecular docking to prioritize repurposing candidate compounds. We identified two clinical CB1 antagonists, MePPEP and otenabant, and the statin drug atorvastatin as promising repurposing candidates based on their high predicted potency and binding affinity toward MDM2. Interactions with the related proteins MDM4 and BCL2 suggest these compounds may enhance p53 restoration through multi-target mechanisms. Quantum mechanical (ONIOM) optimizations and molecular dynamics simulations confirmed the stability and favorable interaction profiles of the selected protein–ligand complexes, resembling that of navtemadlin, a known MDM2 inhibitor. This multiscale, accuracy-boosted workflow introduces a novel data-curation strategy that substantially enhances AI model performance and enables efficient drug repurposing against challenging cancer targets.

## 1. Introduction

**Figure 1:**
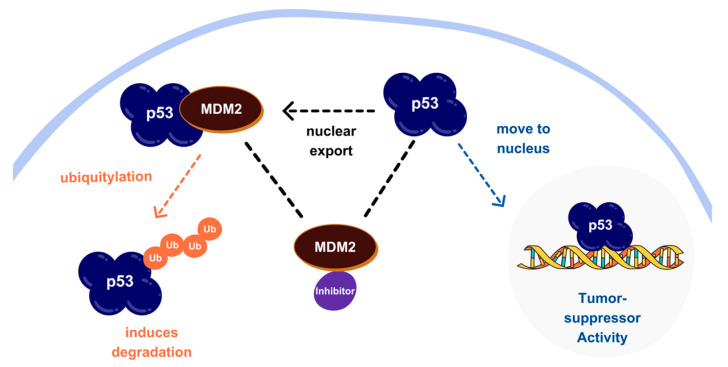
MDM2–p53 regulatory pathway. MDM2 overexpression induces p53 degradation; the inhibitors disrupt this interaction, restoring p53 function.

Over 20 million cancer cases are forecasted globally in 2025, with a ~50% mortality rate, making it one of the leading causes of death [1,2]. Cancer, marked by uncontrolled cell growth and proliferation, encompasses diverse diseases driven by dysregulated cellular pathways. Among the key players in these pathways is the MDM2 protein, a crucial negative regulator of the tumor suppressor p53. Figure 1 shows an illustrative scheme of the MDM2-p53 relationship. In many cancers, MDM2 is overexpressed, suppressing p53 activity and allowing uncontrolled cell proliferation [3,4,5,6,7]. Due to its pivotal role, MDM2 has been recognized as an attractive target for cancer therapy [8].

**Figure 2:**
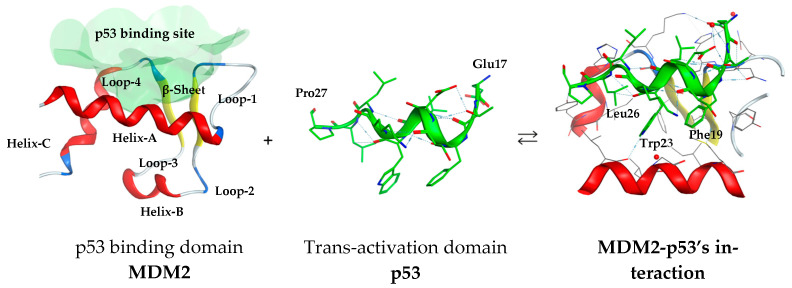
Structural depiction of the MDM2-p53 interaction. MDM2’s N-terminal domain forms a hydrophobic cleft accommodating the α-helical p53 peptide. Key p53 residues—Phe19, Trp23, and Leu26—insert into the pocket, driving the interaction through hydrophobic and π-stacking contacts.

Structurally, MDM2 comprises several functional domains, with its N-terminal 47 residues forming a half-spherical pocket composed of distinct secondary structures. Figure 2 illustrates the 3D structures of MDM2 from a Helix-A perspective at the front. The domain features a deep hydrophobic cleft that facilitates binding to the trans-activation domain of p53, which eventually leads to the inhibition of transcriptional activity. The p53 peptide binds as an amphipathic α-helix, with 10 key residues showing an interaction with the cleft. Notably, three residues of p53—Phe19, Trp23, and Leu26—are known to interact strongly with MDM2 [9,10]. The well-defined architecture of this site enables the rational design of small-molecule inhibitors that mimic p53 binding.

Nearly three decades after its structural discovery, scientists have revealed promising classes of small molecules inhibiting MDM2 [11], specifically targeting the p53 binding domain. These include compounds such as nutlins (cis-imidazoline), spiro-oxindoles, isoquinoline, piperidine/-one, and pyrroloimidazolone, several of which have advanced to clinical trials [12,13,14,15,16,17,18,19,20]. These clinical inhibitors and their known potency against MDM2 are listed in Table 1, while their chemical structures are provided in Supporting Information S1. Analysis of these compounds suggests a common set of chemical features for effective MDM2 inhibition: (i) at least two aromatic systems to facilitate π interactions; (ii) an N-heterocyclic ring, often a lactam, that typically interacts with the aromatic residues in MDM2; and (iii) a 2–3 atom linker which connects adjacent aromatic systems, ensuring an optimal spatial arrangement for binding. Understanding the structural preferences provides valuable insights for the rational design of new MDM2 inhibitors.

Despite the advancement of several clinical candidates, no MDM2-targeting drug has yet reached the market. This shortfall underscores the long, complex, and costly nature of drug discovery and development, particularly in oncology, where the attrition rate can reach up to 95% [21,22,23]. Notably, early-stage drug discovery alone contributes to about a quarter of the total costs [24]. Hence, there is an urgent need to find a robust approach for streamlining the selection of the best molecular candidate to reduce the high failure rate. Recent studies have revealed game-changing digital strategies utilizing data-driven methods such as drug repurposing and machine learning [25,26,27].

Drug repurposing, also known as drug repositioning, stands out as an innovative strategy within drug discovery and development. It leverages the known pharmacological properties and safety profiles of approved or investigational drugs to expedite the development of treatments for different diseases. By bypassing early-stage research and pre-clinical testing, repurposed drugs can swiftly progress to clinical trials, potentially bringing new therapies to patients more rapidly [28]. Despite its advantages, drug repurposing faces challenges such as regulatory approval processes and the need for robust data integration and validation methods [29]. Nevertheless, this approach remains superior for addressing urgent health needs, such as lethal prevalent diseases, pandemics, or rare diseases, where rapid therapeutic development is critical [30,31].

The rise of artificial intelligence (AI), particularly in the realm of machine learning (ML), has had a profound impact on the field of drug discovery. AI-driven algorithms are now being used to design new molecules with desired properties, exploring uncharted chemical spaces [32]. These techniques are mainly employed to build quantitative structure–activity relationship (QSAR) models of bioactivity, and determine the ADMET (absorption, distribution, metabolism, excretion, and toxicity) characteristics of drug candidates [33]. The combination of rapid predictive capabilities and access to vast chemical and biological databases has transformed high-throughput screening into a more cost- and time-efficient process.

On the hunt for effective MDM2 inhibitors, several notable prior studies have reported repurposed small molecules capable of disrupting the MDM2–p53 interaction. For example, Wayne et al. (2012) computationally predicted promising inhibitors from an FDA-approved drug dataset using similarity-based quantification towards nutlin-3a, highlighting the hypertension and chest pain drug S-bepridil as the top hit [34]. Similarly, a porphyrin-based actinic keratosis drug, ALA-protoporphyrin IX, was found through in silico investigation to disrupt the MDM2-p53 interaction, indicating its repurposing potential [35]. Subsequently, in 2021, ospemifene was also computationally identified as a potential dual inhibitor of MDM2-p53 and MDM4-p53 interactions [36]. More recently, by ML-based IC_50_ screening, the antihistamine cetirizine was found to have the potential to inhibit MDM2, thereby activating the p53 pathway and exhibiting antiproliferative effects on glioblastoma and neuroblastoma cell lines [37]. Additionally, nintedanib, an approved treatment of idiopathic pulmonary fibrosis, has been identified as a dual inhibitor of MDM2 and MDM4, disrupting their interaction with p53 and leading to growth inhibition in p53-positive cancer cells [38]. The chemical structures of these proposed MDM2 inhibitors are provided in Supporting Information S1.

Although numerous efforts have been made to identify potential inhibitors, a major ongoing challenge lies in the accuracy of methods used to predict repurposed molecules, particularly those leveraging AI/ML approaches, where consensus results remain elusive. It is well established that the quality of training data is directly linked to model performance [39]. Poor quality or biased datasets can lead to flawed models with limited clinical applicability, as seen in some of the COVID-19 detection models that failed due to biases in the datasets [40]. To address this, a crucial objective of our study was the rigorous preprocessing of data prior to training. We developed and implemented a selective data-cleaning algorithm to retain the most representative molecular data relevant to bioactivity. This algorithm was designed to reduce noise and improve the predictive accuracy of our ML-based virtual screening model.

Our study integrates meticulous data handling, drug repurposing strategies, and AI/ML methodologies to identify novel small molecules capable of disrupting MDM2-p53 interaction. In addition, we examine other relevant receptors, such as MDM4 and BCL2, for their potential inhibitory effects to restore tumor-suppressive functions, ultimately contributing to improved cancer therapies. We also benchmark our proposed hits against clinical MDM2 inhibitors to assess their comparative efficacy.

Finally, we explore the distinct chemical properties of the screened compounds using molecular docking, molecular dynamics (MD), and hybrid quantum mechanics/molecular mechanics (QM/MM) simulation. The insights gained from this in silico investigation aim to support the development of effective and affordable drugs targeting MDM2. With the knowledge of the target biomolecules, our proposed data handling pipeline may also serve as a new standard for high-throughput screening in the discovery of other therapeutic modules.

## 2. Results and Discussion

### 2.1. Optimized Data and ML Model for Virtual Screening

Robust data handling and cleaning are essential for reliable and reproducible ML-assisted drug design. Initially, standard preprocessing steps were applied, including null value removal, normalization (converting IC_50_ to pIC_50_), duplicate elimination to maintain chemical diversity, and SMILES standardization for consistent molecular representation. These steps help reduce bias, improve predictive accuracy, and ensure consistency across cheminformatics analyses. Using a cleaned training dataset, we first compared the performance of 10 regression-based ML models utilizing a collection of 3419 MDM2 inhibitor SMILES (D1_std) with their pIC_50_ values as targets. Table 2 presents the performance of each model following a five-fold cross-validation.

The deep learning model implemented via Chemprop [41], which leverages a graph convolutional neural network (GCNN) [42], outperformed all other models by achieving the highest R^2^ and the lowest RMSE values. The superior performance of GCNN can be attributed to the availability of a large dataset, which is a crucial factor for the effective training of deep learning models. In the context of structure–activity relationships, the dataset size in this study falls within the higher range (thousands), which supports the use of more complex algorithms like GCNN. However, it is important to note that the optimal dataset size for model performance can vary significantly depending on the specific application. Another reason is the use of neural fingerprint or learned molecular representation. When Chemprop predicts properties, it does not rely on pre-calculated fingerprints (like Morgan, MACCS, etc.). Instead, it simultaneously learns the optimal way to encode the molecule into a fingerprint that best explains the property data, thus capturing task-specific chemical insights. Given its superior performance, GCNN via Chemprop will be utilized for the subsequent analyses of this study.

To further improve performance, hyperparameter tuning was conducted. Specifically, different epoch hyperparameters were tested to examine the performance of the Chemprop-based predictor. It is well documented that training a model for too few epochs may lead to underfitting, where the model fails to capture important patterns in the data. Conversely, training for too many epochs may lead to overfitting, where the model memorizes the training data and fails to generalize to unseen data. The optimal epoch value was determined to be 70, balancing computational cost and model performance (Figure S4a). Other hyperparameters were optimized using a Bayesian search function to ensure their optimal contribution to the model.

The model’s generalization towards a new dataset was evaluated using the scaffold split approach. In contrast to the random split, scaffold splitting partitions the dataset based on structural preferences so that the same molecular scaffold does not appear in multiple splits. This approach tests the model’s ability to handle structurally diverse, previously unseen compounds. The results show that the model may handle this situation well, as evidenced by the minimal increase in error ($\Delta \bar{RMSE}$ = 0.09) when applying scaffold splitting, as shown in Figure S4b.

The dataset optimization involved enrichment, filtering, and selective cleaning, with their respective impacts summarized in Table 3.

**Figure 3:**
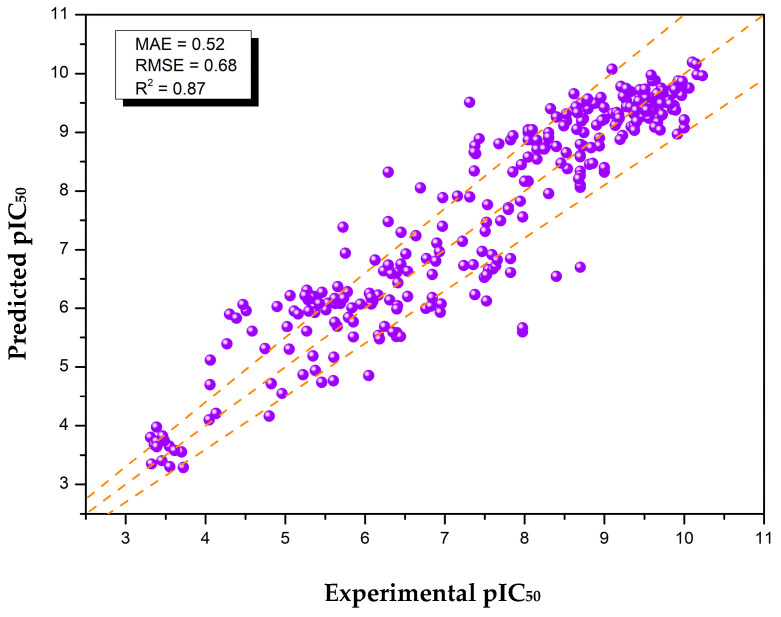
Parity plot showing the optimized deep learning model’s final performance on the unseen test dataset. Each purple circle represents a molecule′s predicted (y-axis) versus experimental (x-axis) pIC_50_ value. The central-dashed-orange ideal line indicates perfect prediction accuracy (y = x), while the upper and lower dashed lines represent a ±0.5 deviation. Model performance metrics (MAE, RMSE, and R^2^) are provided.

The results indicate that the combination of extra data points and optimization approaches has significantly reduced the model’s RMSE, leading to a 21.6% performance improvement. The additional data provide more training examples, effectively enhancing the learnability. Implementing filters such as molecular weight thresholds to exclude overly bulky (>750 Da) or overly simplistic molecules (<100 Da) helps eliminate outliers that could introduce bias into the model’s learning process. SC implementation has proven to selectively keep the best examples from a pool of data duplicates with the knowledge of the assay procedure, which in turn reduces bias. With SC incorporated, the RMSE reduced to 0.58, reflecting an improvement in the model’s predictive accuracy. Moreover, to the best of our knowledge, the SC pipeline combined with the utilization of publicly available databases and hyperparameter tuning achieved the highest R^2^ of 0.87 for the pIC_50_ predictive model targeting the MDM2 protein (Figure 3).

The SC pipeline helps resolve ambiguity when selecting the most appropriate data entry for an ML model, particularly in cases where a single molecule has multiple bioactivity values, which is often the case when extracting data from public databases. Based on our observations, the presence of multiple bioactivity values may be attributed to two main factors: (i) different assay procedures being used to generate the data, and (ii) different stereoisomers of the compound, as the specific stereoisomer corresponding to each bioactivity value has not been explicitly identified or specified. Nutlin-3a bioactivity is a notable example of issue (i), as a well-studied molecule with 10 reported pIC_50_ entries toward MDM2 from the ChEMBL database. Table S2 shows the variation in the assay types being used and their respective pIC_50_ output. For issue (ii), we identified a proprietary MDM2 inhibitor in Figure S2 with two stereocenters that have five reported IC_50_ values despite originating from the same assay procedure and source. This highlights how stereoisomerism can introduce discrepancies in bioactivity data.

**Figure 4:**
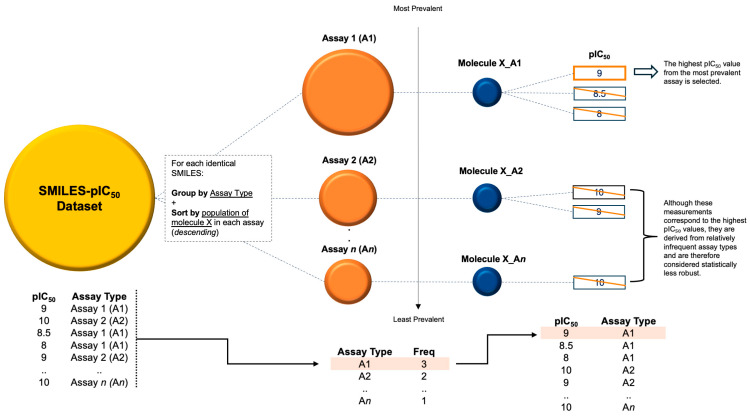
The schematic diagram of the SC algorithm illustrates its two-layer verification process designed to identify the most reliable bioactivity value, which properly represents the drug target’s bioactivity. The example represents a modest case of how selective cleaning selects the most robust measurement from a hypothetical “Molecule X” with multiple pIC_50_ values.

Previous approaches for choosing the representative bioactivity from multiple entries include the following: (1) random selection, (2) average, (3) choosing the highest value, and (4) selecting the most recent entry. Out of the four, choosing the highest pIC_50_ is often preferred since it reflects the best capabilities of the molecule. However, this approach may also lead to unintended bias if the data originate from an infrequently used assay procedure. Statistically, this would make the value an outlier among the other values, making it less reliable. The SC algorithm addresses this issue by selecting the highest pIC_50_ from the most prevalent assay, as illustrated in Figure 4, ensuring a more consistent and meaningful representation of the molecule’s bioactivity. Despite the improvement in model performance from SC by prioritizing common high-quality assays, we recognize that assay design, reagent differences, and protocol variations can still introduce residual bias. Future work could be directed to improve SC by exploring assay-specific quality metrics and including them in the ranking step to further refine the selection of representative bioactivities.

### 2.2. Integrated Virtual Screening Hits for Potential MDM2 Inhibitors

After the model was fully optimized, more than 24,000 molecules from the D2 dataset were deployed to predict their activities toward MDM2. This dataset was thoughtfully collected from every bioactive compound listed in the four well-established databases, ensuring a diverse chemical space beyond commercially available drugs while focusing on biologically active compounds with empirical validation. The repurposing candidates spanned from preclinical- to commercial-stage compounds, including some previously retracted drugs.

**Figure 5:**
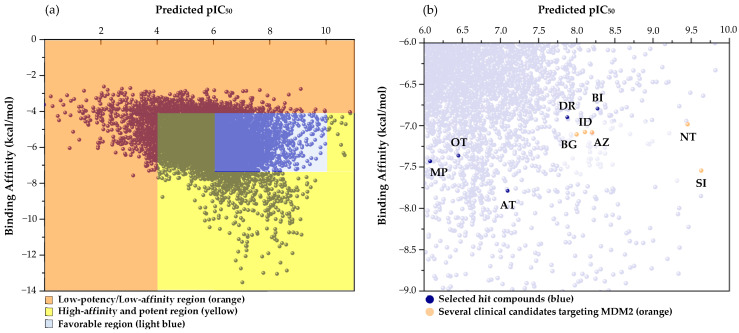
Distribution of predicted pIC_50_ and binding affinity from virtual screening. (**a**) Predicted pIC_50_ vs. docking score (kcal/mol) for all D2 compounds, highlighting three regions: low-potency/low-affinity (orange), high-affinity/potent (yellow), and favorable hits (light blue). (**b**) Enlarged view of the favorable region showing the top five selected hits—MePPEP (MP), otenabant (OT), atorvastatin (AT), BIRT-2584 (BI), and drinabant (DR)—alongside clinical MDM2 inhibitors (orange circles). Clustering of hits with known inhibitors supports the screening strategy.

On selecting the potential hits, the calculated properties were benchmarked against the known MDM2 inhibitors’ predicted activity and structural similarity. In Figure 5a, the predicted pIC_50_ is plotted against the calculated binding affinity, creating a two-dimensional map of potential inhibitors from the D2 database. The orange region comprises compounds with relatively low potency (lower pIC_50_ values) and weaker receptor interactions (less favorable binding affinities). Such molecules are unlikely to disrupt the MDM2 binding interface effectively and are thus deprioritized for further development.

Meanwhile, the yellow region spans compounds with moderate-to-high potency and binding affinity but which still fail to meet the criteria for being promising inhibitors. Some compounds in this region resemble the low-potency/low-affinity scenario seen in the orange region, while others exhibit exceptionally high potency or affinity, which may be associated with unfavorable toxicity profiles (e.g., off-target effects or lethal outcomes). Consequently, even though these molecules appear potent at first glance, they may not be suitable candidates for drug development without extensive safety evaluations.

In contrast to the lower-performing regions, the blue-shaded area (spanning pIC_50_ values of 6.0 to 10.0 and binding affinities ranging from −9.0 to −6.0 kcal/mol) highlights a subset of compounds that exhibit both favorable potency and sufficient receptor interactions. Many known MDM2 inhibitors cluster within this domain, suggesting that it delineates a “sweet spot” where chemical entities balance adequate p53 reactivation potential and manageable off-target effects. Focusing on molecules within this region allows the screening process to prioritize candidates with a higher likelihood of exhibiting optimal therapeutic indices.

This screening approach also highlights the advantages of utilizing a regression-based ML model over a binary classification framework. Unlike classification models that rigidly categorize compounds as either active or inactive, a regression model provides a continuous spectrum of predicted pIC_50_ values, allowing for a more refined assessment of potential inhibitors. This flexibility enables the identification of compounds that may not meet strict binary cutoffs but still possess promising activity trends. Moreover, the regression model facilitates clustering analysis within the dataset, helping to delineate compounds that fall into the optimal potency and binding affinity range.

To further refine the selection, strict structural criteria were imposed in addition to these calculated properties. Specifically, candidate molecules were required to possess halogen bond donors (or equivalent groups with sigma holes), a modest number of aromatic rings (two to four), and a constrained spatial arrangement to avoid overly large or flexible scaffolds. Potential hits were also filtered to exclude peptides, nucleotides, fatty acid derivatives, and molecules bearing extensive aliphatic chains. These combined criteria helped eliminate compounds prone to metabolic liabilities or unsatisfactory pharmacokinetic profiles, resulting in a focused set of top-ranking hits for subsequent validation.

The top five hits (H1) that matched the criteria, namely MePPEP (MP), otenabant (OT), atorvastatin (AT), BIRT-2584 (BI), and drinabant (DR), were picked from the same cluster with the top clinical inhibitors, as depicted in Figure 5b. The calculated properties, mechanisms of action, and chemical structures of H1 compounds are tabulated in Table 4.

**Figure 6:**
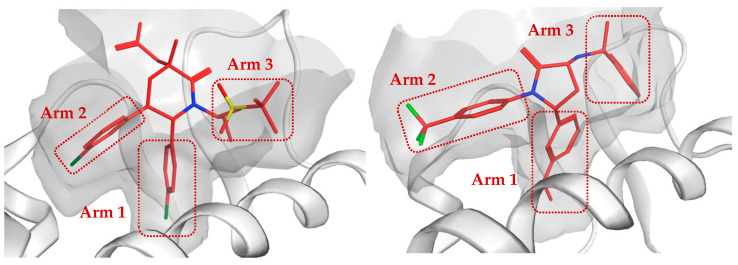
Structural similarity between MDM2-NV (**left**) and MDM2-MP (**right**).

Interestingly, three CB1 antagonists, MP, OT, and DR, emerged concurrently as the top hits. This observation may be attributed to the structural similarity between the conformations of MDM2 ligands and those of CB1. Upon examining the binding modes of the CB1 antagonist, MP, towards the MDM2 pocket, it showed a strong conformational correlation with MDM2-NV, as shown in Figure 6. CB1 antagonists have three moieties oriented towards different directions, previously termed arm 1, arm 2, and arm 3 [43]. This pattern can also be observed in MDM2 inhibitors, which show comparable geometry.

### 2.3. Affinity and Site-Selectivity Validation of the Top Hits Using Redocking Simulations

To confirm the binding capabilities of the five selected H1 hits, we performed redocking simulations against MDM2 and examined their off-target potential with MDM4 and BCL2. Table 5 summarizes the consensus docking results across three different software packages (MOE 2022.02, AutoDock Vina 1.2.5, and GOLD 2024.1.0). Overall, the negative binding affinity values for MDM2 underscore that all five compounds retain a plausible capacity to engage the receptor’s primary binding pocket (p1). Notably, most hits showed a slight preference for pocket p1 over the secondary site (p2), as indicated by positive “p1−p2” differences (e.g., 0.5 kcal/mol for MP and OT), suggesting higher selectivity.

Beyond MDM2, we also explored the ligands’ affinities for MDM4 and BCL2, two proteins implicated in the complementary pathways that promote cancer cell survival. Indeed, co-inhibition of these targets alongside MDM2 has been reported to yield synergistic therapeutic benefits [44,45,46]. Among the H1 compounds, AT exhibited an especially strong binding toward MDM4 (−11.1 kcal/mol), while OT showed a notable affinity for BCL2 (−7.4 kcal/mol).

Despite confirming favorable docking scores and toxicity profiles (see Table S3), we ruled out BIRT-2584 (BI) and drinabant (DR) based on their relatively less desirable properties, including their suboptimal site-selectivity or lower predicted potency. Consequently, we designated MP, OT, and AT as the final H2 hits for subsequent investigation. These compounds consistently exhibited robust binding to MDM2’s p1 pocket across all scoring platforms, alongside having promising off-target profiles that could potentially bolster anticancer efficacy.

### 2.4. Analysis of Protein–Ligand Interactions in Optimized MDM2-H2 Compound Complexes

To explore how the selected H2 compounds interact with the MDM2 binding pocket, QM/MM (ONIOM) simulations were employed. These simulations provide insight into the electronic polarization effects of ligands while allowing the receptor to adopt flexible conformations. Adding strict optimization processes and flexibility is considered more reliable in molecular recognition [47].

**Figure 7:**
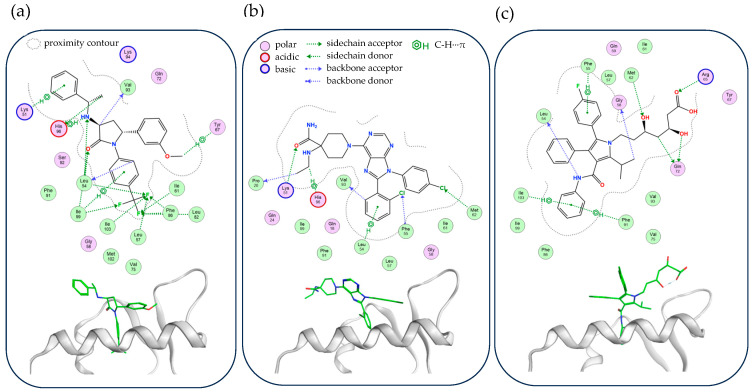
Molecular recognition of optimized MDM2–ligand complexes: (**a**) MP, (**b**) OT, (**c**) AT complexes, represented in 3D pocket view and as the isolated ligand.

The optimized geometries revealed stable conformations, the key contact residues that isolate the ligands, and their intermolecular interactions (Figure 7). The conformation follows the pattern from Figure 6, where the ligand’s three arms are directed to similar MDM2 pocket regions.

The overall distribution and populations of the interactions aggregated by selected top residues are summarized in Table 6. Ligand interactions were categorized by their energy using Extended Hückel Theory (E_EHT_) values; these were collectively denoted by +/++/+++, while the sign of weak interactions (−1.0 < E_EHT_ < −0.1 kcal/mol) was denoted as ‘+’, moderate interactions (−3.0 < E_EHT_ < −1.0 kcal/mol) as ‘++’, and strong interactions (E_EHT_ < −3.0 kcal/mol) as ‘+++’. Insignificant interactions (E_EHT_ > −0.1 kcal/mol) were marked with ‘-’. From the interaction distribution, Leu54 and Ile99 stood out as key contact residues, reinforcing their important roles in MDM2 recognition. Notably, these residues also serve as structural anchors for ligand stabilization in native p53 binding and in complex formation with NV.

The detailed NCI strengths calculated are listed in Table S5. The interaction analysis reveals a diverse set of hydrogen bonds, C-H···π interactions, C-H···O interactions, and halogen bonding. In terms of interaction strength, hydrogen bonding plays a dominant role, with the most stabilizing interactions observed in MDM2-AT, where Arg65 and Leu54 exhibit strong N-H···O hydrogen bonds that significantly enhance ligand binding. OT also forms highly stabilizing hydrogen bonds, particularly with Lys51. In contrast, MP mostly interacts with Arg65 and Ile61 through a combination of C-H···O, C-H···F, and C-H···π interactions, reflecting a diversified but comparatively weaker interaction profile compared to those of AT and OT.

Among the three ligands, AT demonstrates the strongest interaction with MDM2, as evidenced by its highly stabilizing hydrogen bonds, in addition to its extensive C-H···π interactions with Phe55 and Phe91. OT also forms robust interactions, particularly with Lys51, Leu54, and His96. With additional reliance on Cl···O halogen bonding through Leu57 and Phe55, it introduces a different stabilization mechanism that may influence its binding specificity. MP, while effectively engaging key residues, exhibits slightly lower overall binding energies due to its increased reliance on C-H···O interactions rather than direct hydrogen bonds.

When comparing which compound best mimics the binding pattern of p53 (see Supporting Information S5.2), AT and MP appear as the closest structural and functional analog. The MDM2-p53 interaction is characterized by key hydrogen bonds and C-H···π interactions, particularly involving Leu54 and Gln72, which are also primary binding sites in MDM2-AT/MP stabilization.

On the other hand, NV exhibits a stronger halogen bonding component, particularly for Cl···O and chalcogen bonding C-H···S interactions, along with π-stacking centered around His96 and Lys94 (see Supporting Information S5.3). This binding mode is more similar to OT, which also relies on halogen bonds and interactions with His96.

Thus, AT displays the strongest interaction with MDM2, and along with MP, it also resembles the p53 binding mechanism, whereas OT aligns more closely with NV’s interaction pattern. This distinction highlights the potential for AT/MP to act as a functional p53 mimic while suggesting that OT might offer an alternative stabilization mechanism that leverages halogen interactions for MDM2 inhibition.

### 2.5. Binding Stability and Deep Pocket Insertion of MDM2-H2 Compound Complexes from MD Simulations

**Figure 8:**
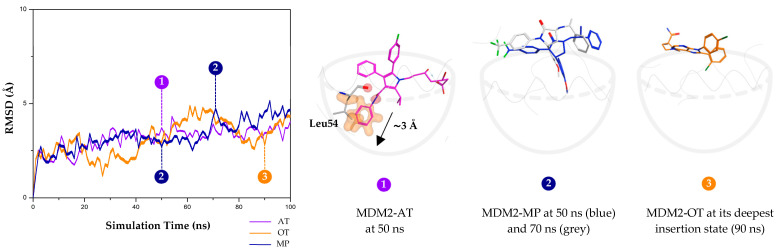
Analysis of ligand binding stability by RMSD plot and snapshot visualization from MD simulation trajectories.

MD simulations were performed to confirm the stability of the ligand binding within the protein active site over a 100 ns timeframe. Binding stability was assessed by monitoring structural fluctuations through the root-mean-square deviation (RMSD) plot shown in Figure 8. RMSD is a commonly used metric to evaluate conformational changes over time relative to the initial structure.

Among the three ligands, AT exhibited the least RMSD fluctuation, indicating its superior stability compared to other ligands. Trajectory visualization of MDM2-AT at 50 ns revealed key interactions, particularly hydrogen bond and π interactions with Leu54 residue, corroborating the findings from optimized geometry analysis. MP also showed a relatively stable trend, despite significant movement around 70 ns, likely due to the movement of the ligand away from the deeper cleft (Figure 8). However, we observed that OT inhibition showed significant fluctuations across multiple time frames, which suggests reduced binding stability.

The nature of OT’s instability would be the result of the rigidity of its aromatic moiety, which restricts deeper cleft insertion. Figure 8 shows MDM2-OT at its deepest insertion state (90 ns). Unlike AT, MP, or even clinical inhibitors like NV, which feature flexible linkers enabling better positioning of aromatic groups into the binding cleft, OT lacks such adaptability. This insertion towards the deeper cleft is driven by affinities towards residues Leu54 (Helix A) and Phe86 (Helix B), a critical factor in enhancing binding affinity. We observed the deep insertion from AT at around 3 Å, which is comparable to that observed with NV (see Supporting Information S5.3), highlighting the importance of this feature in the design of potent MDM2 inhibitors.

## 3. Methods

### 3.1. Training Dataset Preparation

This study begins by collecting a diverse dataset of known MDM2 inhibitors with their IC_50_ potency sourced from the ChEMBL database of bioactive molecules to build the training dataset (D1) [48]. After gathering the inhibitors, we constructed a data frame using the Pandas 2.1.4 library [49] in the Python 3.11.4 [50] environment to perform data preprocessing. To ensure robust model evaluation, molecular scaffold splitting was applied, where 10% of the D1 was set aside as the final testing dataset (D1_testing). The complete data preparation and preprocessing pipeline is accessible at our GitHub Repository (https://github.com/firdauusakmal/MDM2pipeline, accessed on 5 July 2025).

**Figure 9:**
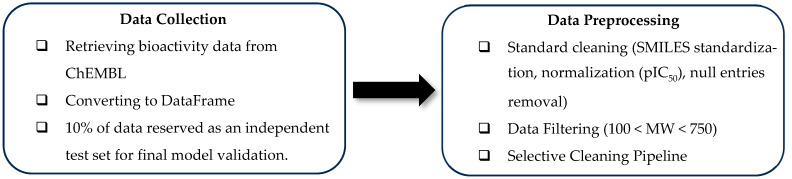
Schematic diagram of data preparation and preprocessing workflow.

The data preprocessing workflow consisted of three sequential steps to enhance data quality: (1) Standard cleaning, including standardization of molecular structures, conversion to pIC_50_ values, and removal of null entries. The molecular data, in SMILES representation, were standardized using a Python library, MolVS, which operates within the RDKit chemistry framework [51]. (2) Filtering of molecular data was performed based on molecular weight (MW) criteria (100 < MW < 750) to remove outliers. (3) Selective cleaning (SC) was implemented as depicted in Figure 9. For the SC pipeline, it started by grouping the bioactivity data based on the most populated assay procedure in descending order; then, sorting the bioactivity values within each group independently; and lastly, keeping the highest value of each molecule at the top as the maximum bioactivity and selectively removing duplicate or redundant entries. The entire process was executed using the pandasql 0.7.3 library [52]. Finally, we systematically recorded the impact of stacking preprocessing steps (standard cleaning, filtering, and SC) on model performance enhancement.

### 3.2. ML Selection and Optimization

For selecting the most effective ML models, we evaluated the performance by following these steps:

- Feature Extraction: We extracted a set of 300 two-dimensional molecular descriptors—computed via Chemprop’s integrated RDKit feature generator—for use as input features in our scikit-learn models.
- Data Splitting: The dataset was divided into training (D1_training) and validation sets, with 20% allocated for validation.
- Model Training: Various ML models were trained using the scikit-learn 1.4.0 package [53], including k-nearest neighbor, decision tree, random forest, AdaBoost, XGBoost, gradient boosting, histogram gradient boosting, stochastic gradient descent, and multi-layer perceptron. Additionally, deep learning models were trained using ChemProp 1.6.1 [41], leveraging ChemProp’s neural fingerprints and pIC_50_ target values.
- Hyperparameter Optimization: Each model underwent hyperparameter tuning to enhance performance, and different data optimization procedures were compared using the validation set.
- Final Evaluation: The best performing models were tested on the independent D1_testing dataset to assess their predictive accuracy.

### 3.3. Repurposing Dataset Preparation

The repurposing dataset (D2) was established from molecules with known therapeutic indications. These series of molecules were collected from specialized bioactive sets curated by different databases, including ChEMBL, PubChem, DrugCentral, and DrugBank [54,55,56]. To ensure consistency, all molecules were standardized using the same protocol as the D1 for handling SMILES representation.

### 3.4. ML-Based Virtual Screening

The collection of SMILES in the D2 dataset was then deployed to the best performing ML model, and the pIC_50_ of each molecule was predicted.

### 3.5. Structure-Based Virtual Screening (SBVS)

We employed molecular docking simulations as part of the SBVS process to identify potential MDM2 inhibitors from D2. First, the crystal structure of the MDM2 protein (PDB ID: 6Q9L, 1.13 Å) [57], retrieved from the RCSB Protein Data Bank [58], served as the target receptor for docking studies. The protein was initially prepared to strip water molecules from the system and adjust the protonation state of the residue at pH 7.4. The simulations were then performed using MOE 2022.02 [59], where the D2 compounds (in SDF format) were docked into the MDM2 binding site (reference ligand placement) to predict their binding affinities and orientations. The selected placement methodology was ‘Triangle Matcher’, and all receptor atoms were held fixed during the refinement. The top hit compounds (H1) were determined from the virtual screening results and from benchmarking toward existing MDM2 inhibitors.

### 3.6. Redocking Analysis and Toxicity Prediction

The H1 compounds were redocked against MDM2 to confirm their site-selectivity and affinities. The simulations were initially performed to compare the affinity of the ligands toward the p53 binding site (p1) and another well-defined pocket detected by MOE (p2). Then, the consensus docking was performed using multiple platforms: MOE 2022.02, Autodock Vina 1.2.5 [60,61], and GOLD 2024.1.0 [62], providing multiple scoring functions to reduce platform-specific bias [63]. The scoring functions that were used on each software, respectively, were GBVI/WSA dG, X-score, and ChemPLP. More detailed information on the selection of the binding site is available in Supporting Information S3.1.

The H1 compounds were also simulated against BCL2 (PDB ID: 8HTS, 1.25 Å) [64] and MDM4/MDMX (PDB ID: 6Q9Y, 1.2 Å) [57] for the off-target affinities. Additionally, multiple toxicity endpoints were predicted using ProTox-3.0 [65], including acute oral toxicity (LD_50_), hepatotoxicity, cardiotoxicity, carcinogenicity, and mutagenicity. The set was further shortlisted upon redocking and off-target docking completion to form the final hit compounds (H2).

### 3.7. ONIOM Simulation

To refine binding interactions between H2 molecules and MDM2, geometry optimizations were performed using the “Our own N-layered Integrated molecular Orbital and molecular Mechanics” (ONIOM) method in Gaussian 16 [66] integrated with the MOE interface. Each system was divided into two layers, where the ligands (high-level layer) were optimized under the ωB97X-D functional [67] with the 6–31G* basis set [68]. The remaining receptor (low-level layer) was treated using molecular mechanics (MM) with the AMBER10 force field [69]. The optimized geometries of protein-–ligand complexes were analyzed to compare the binding mode and non-covalent interactions (NCIs). The energy components were calculated using Extended Hückel Theory (EHT) [70] as implemented in the MOE 2022.02 software.

### 3.8. Molecular Dynamics Simulations

MD simulations in MOE were performed to explore the dynamic behavior of MDM2-hit compounds (H2). The Nosé–Poincaré–Andesen (NPA) [71] integrator was chosen for its high precision and sensitivity in modeling for small molecules and compact systems [72]. Initially, the complex was solvated in aqueous KCl 0.15 M (ρ = 1.022 g/cm^3^) in a 50.5 × 40 × 40 Å^3^ cell. The stable system from the previous simulation was subjected to heating (100 ps), NVT (100 ps), and NPT (200 ps) ensembles, respectively, at 310 K and 101.3 kPa to match the cellular environment. The production MD simulations were then performed to simulate the time evolution of the complex for 100 ns with a time step of 2 fs to capture the time evolution of the complex.

## 4. Conclusions

In this study, we developed a machine-learning-assisted virtual screening pipeline optimized for identifying potential MDM2 inhibitors through meticulous data curation, deep learning model refinement, and structure-based virtual screening. The integration of a selective cleaning (SC) algorithm boosted the accuracy of pIC_50_ predictions, reducing the RMSE by 21.6% and achieving an R^2^ of 0.87, thereby enhancing the reliability of the predictive model.

Applying the optimized model to a repurposed drug library of over 24,000 molecules, we identified five promising candidates, namely MePPEP, otenabant, atorvastatin, BIRT-2584, and drinabant, which exhibited strong binding affinities and favorable physicochemical properties. Further refinement through consensus docking and off-target screening narrowed the selection to three final hits (MePPEP, otenabant, and atorvastatin). These compounds demonstrated high selectivity for MDM2 and interaction profiles comparable to clinical inhibitors.

Further non-covalent interaction analysis using ONIOM-optimized geometries revealed that all three compounds engaged critical MDM2 residues (Leu54 and Ile99), with atorvastatin displaying the strongest binding affinity through highly stabilizing hydrogen and C-H···π interactions. Molecular dynamics (MD) simulations over 100 ns confirmed the stability of these inhibitors, with atorvastatin exhibiting the most consistent binding, followed by MePPEP, while otenabant displayed higher fluctuations, likely due to its limited deep-pocket engagement.

Overall, our findings provide a validated framework for accuracy-boosted AI-driven drug repurposing and highlight MePPEP, otenabant, and atorvastatin as promising MDM2 inhibitor candidates for further preclinical development. The mechanistic insights into their binding interactions and structure–activity relationships offer valuable directions for lead optimization and the rational design of next-generation MDM2-targeting anticancer therapies.

## Figures and Tables

**Table 1:** Recent progress of clinical small molecules targeting MDM2.

Name	Max. Clinical Phase	Type of Compounds	pIC_50_	Ref
Navtemadlin (NV)	III	Piperidine/-one	9.22	[12]
Idasanutlin (ID)	III	Nutlins	8.22	[13]
Brigimadlin (BG)	III	Spiro-oxindoles	7.92	[14]
Siremadlin (SI)	II	Pyrroloimidazolone	9.64	[15]
Alrizomadlin (AZ)	II	Spiro-oxindoles	8.42	[16]
CGM097	I	Isoquinoline	8.77	[17]
Milademetan	I	Spiro-oxindoles	7.75	[18]
RG7112	I	Nutlins	7.74	[19]
MI-773	I	Spiro-oxindoles	7.00	[20]

**Table 2:** Performance comparison of different regression-based ML models (five-fold cross-validation).

ML Model	RMSE	R^2^
k-Nearest Neighbor	0.84	0.77
Decision Tree	0.99	0.69
Random Forest	0.82	0.78
AdaBoost	0.99	0.69
XGBoost	0.86	0.76
Gradient Boosting	0.81	0.79
Histogram Gradient Boosting	0.81	0.79
Stochastic Gradient Descent	0.93	0.72
Multi-Layer Perceptron	0.79	0.80
Graph Convolutional Neural Network	0.73	0.84

**Table 3:** Performance comparison of the deep learning model following the different steps of the data optimization procedures on D1.

Data Optimization	Number of Data Points	RMSE	R^2^
Standard Cleaning ^a^	1926	0.74	0.81
Standard Cleaning ^b^	3419	0.73	0.84
Standard Cleaning + Filtering	2954	0.64	0.86
Full Optimization (With Selective Cleaning)	2954	0.58	0.87

^a^ 1926 standard-cleaned inhibitors targeting MDM2/CHEMBL5023 (D1_std_1926). ^b^ Extra data points added from inhibitors targeting the MDM2-p53/CHEMBL1907611 protein–protein interaction (D1_std_3419).

**Table 4:** Properties of selected hits from virtual screening of repurposed set (H1).

Molecule Name	Mechanism of Action	Max Clinical Phase	Two-Dimensional Structure	Binding Affinity (kcal/mol)	Predicted pIC_50_
MePPEP (MP)	CB1 antagonist	II	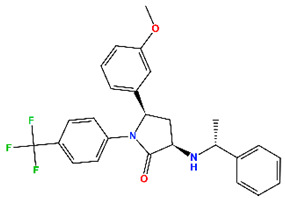	−7.43	6.08
Otenabant (OT)	CB1 antagonist	III	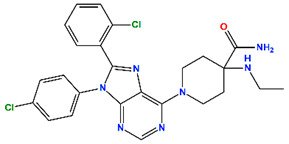	−7.36	6.45
Atorvastatin (AT)	HMG-CoA reductase inhibitor	Approved	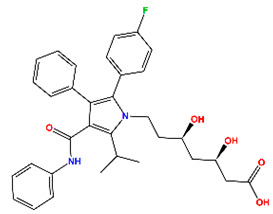	−7.79	7.10
BIRT-2584 (BI)	ITGAL and ITGB2 antagonist	II	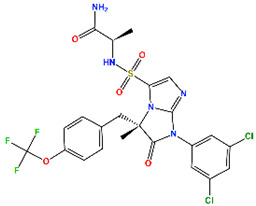	−6.79	8.27
Drinabant (DR)	CB1 antagonist	Preclinical	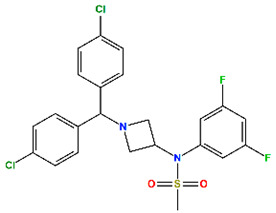	−6.90	7.88

**Table 5:** The calculated docking scores from redocking simulations.

Ligand	Docking Score						
	MOE (kcal/mol)					Autodock Vina (MDM2; kcal/mol)	GOLD (MDM2)
	MDM4	BCL2	MDM2_p1	MDM2_p2	p1−p2		
MP	−9.6	−6.6	−7.6	−7.1	0.5	−7.7	72.7
OT	−9.4	−7.4	−7.5	−6.9	0.5	−8.1	64.0
AT	−11.1	−7.3	−8.4	−8.1	0.3	−7.8	61.4
BI	−10.1	−7.1	−7.4	−7.5	−0.1	−8.0	65.4
DR	−9.3	−6.4	−7.2	−6.8	0.4	−8.0	72.1

**Table 6:** Interaction profiles of top MDM2 residues with H2 compounds MP, OT, and AT. Non-covalent interactions were classified by E_EHT_ energy: weak (−1.0 < E_EHT_ ≤ −0.1 kcal/mol) as ‘+’, moderate (−3.0 < E_EHT_ ≤ −1.0 kcal/mol) as ‘++’, and strong (E_EHT_ ≤ −3.0 kcal/mol) as ‘+++’; insignificant interactions (E_EHT_ > −0.1 kcal/mol) are denoted ‘-’. Leu54 and Ile99 emerge as particularly important contact points, consistent with their roles in native p53 binding and in MDM2–NV complex stabilization.

Residue	MP	OT	AT	Type of Interaction
Arg65	+++	-	+++	Hydrogen bond
Leu54 ^a^	+	+++	+++	Hydrogen bond
Gln72 ^a^	+	-	++	Hydrogen bond
Lys51	-	+++	-	Hydrogen bond
Met62 ^a^	-	+	++	C-H···Cl/C-H···O interaction
Ile61	+++	++	+++	C-H···F interaction
Ile99 ^b^	+	++	++	C-H···π interaction

^a^ involved in MDM2-p53 interaction. ^b^ involved in MDM2-NV interaction.

## Data Availability

To obtain the SMILES and IC_50_ data for the training dataset, the ChEMBL database (https://www.ebi.ac.uk/chembl/, accessed on 29 January 2024) was utilized. The complete data preparation and preprocessing pipeline, including the selective cleaning (SC) algorithm, is available at our GitHub repository: https://github.com/firdauusakmal/MDM2pipeline/ (accessed on 5 July 2025). The repository also contains scripts for ML models implemented using scikit-learn 1.4.0, as well as checkpoint files and configurations for reproducing the deep learning models developed with ChemProp 1.6.1 (https://github.com/chemprop/chemprop, accessed on 20 June 2024). The repurposing dataset was curated from publicly accessible bioactive compound databases, including ChEMBL, PubChem (https://pubchem.ncbi.nlm.nih.gov/, accessed on 21 January 2024), DrugCentral (https://drugcentral.org/), and DrugBank (https://go.drugbank.com/, accessed on 21 January 2024). Crystal structures for MDM2, MDM4, and BCL2 were retrieved from the RCSB Protein Data Bank (https://www.rcsb.org/, accessed on 2 November 2023). Molecular docking was conducted using both commercial and open-source software. Structure-based virtual screening was performed using the licensed MOE 2022.02. Consensus docking employed AutoDock Vina 1.2.5 (open source) and GOLD 2024.1.0 (licensed). ONIOM and MD simulations were carried out within the MOE interface, utilizing licensed versions of Gaussian 16 and the NPA integrator. The inputs for MD simulations are available in the GitHub repository.

## Supplementary Materials

The following supporting information can be downloaded at: https://www.mdpi.com/article/10.3390/molecules30142992/s1; S1. Recent progress of small molecules targeting MDM2; S2. Origins of redundant-entry bias; S3. Hit optimization setup; S4. Optimizing ML model; S5. Non-covalent interaction energetics.

## Abbreviations

The following abbreviations are used in this manuscript:

BCL2	B-Cell Lymphoma-2 Protein
COVID-19	Coronavirus Disease 2019
FDA	The United States Food and Drug Administration
IC_50_	Half-Maximal Inhibitory Concentration
MDM2	Mouse Double Minute 2 Protein
MDM4	Mouse Double Minute 4 Protein
SMILES	Simplified Molecular Input Line Entry System
