# Supplementary material for: Selective Cleaning Enhances Machine Learning Accuracy for Drug Repurposing: Multiscale Discovery of MDM2 Inhibitors
Source: Molecules. 2025 Jul 16;30(14):2992. doi: 10.3390/molecules30142992 (PMC12299252; doi:10.3390/molecules30142992)
Supplement: Supplementary file 1 [file molecules-30-02992-s001.zip › molecules-3716759-supplementary.pdf]

Selective Cleaning Enhances Machine Learning Accuracy for Drug Repurposing: Multiscale Discovery of MDM2 Inhibitors

Mohammad Firdaus Akmal, Ming Wah Wong*

Department of Chemistry, Faculty of Science, National University of Singapore, 3 Science Drive 3, Singapore 117543

Supplementary Materials

Table of Contents

S1. Recent Progress of Small Molecules Targeting MDM2 2

S2. Origins of Redundant-Entry Bias 4

S3. Hit Optimization Setup 5

S4. Optimizing ML Model 6

S5. Non-covalent Interaction Energetics 6

S1. Recent Progress of Small Molecules Targeting MDM2

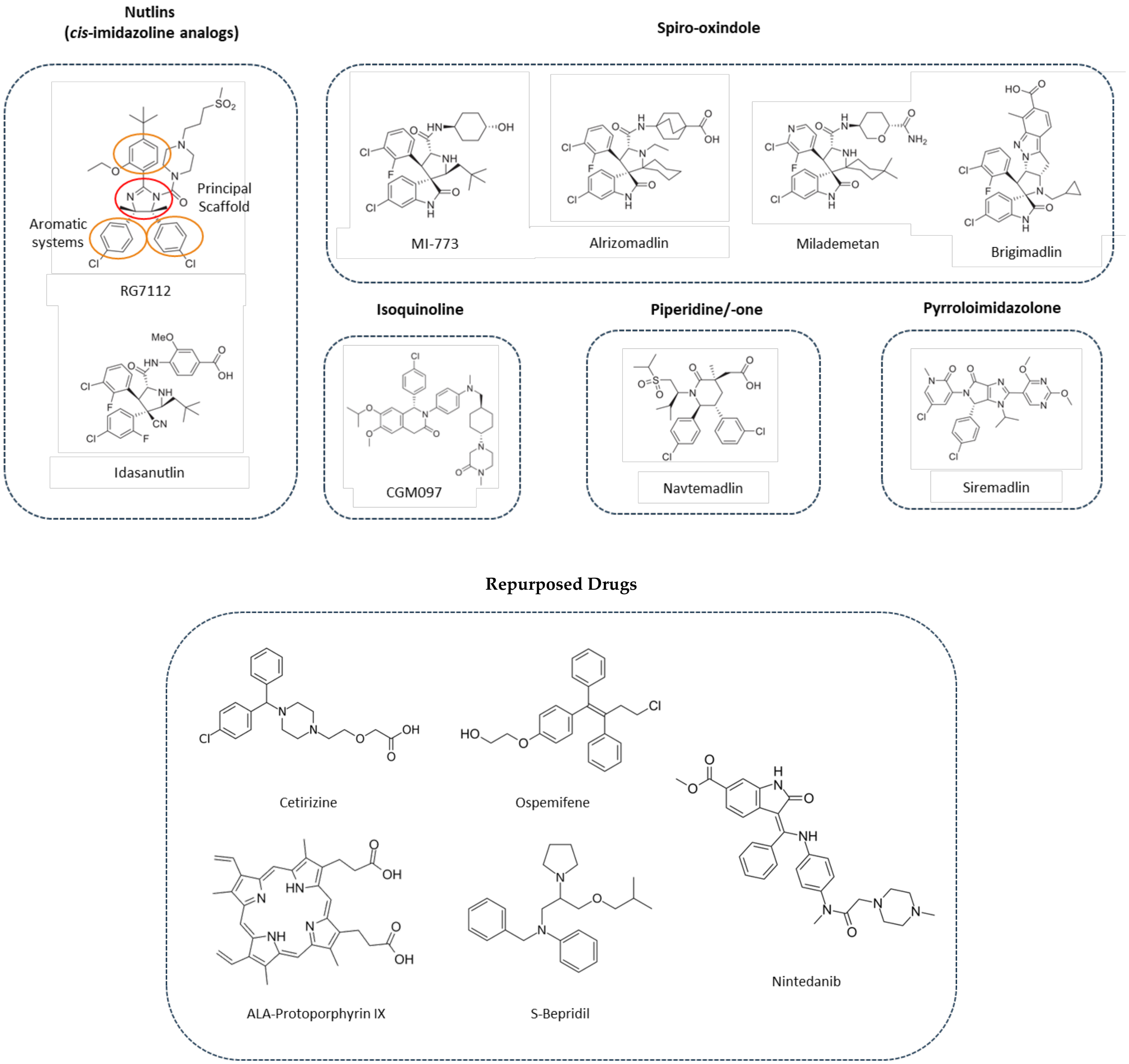

Figure S1. The chemical structure of recent clinical candidates and repurposed drugs targeting MDM2

Table S1. Recent repurposed drugs targeting MDM2

Name	Original Purpose	Screened Database	Docking Score (kcal/mol)	Ref
S-bepridil	angina pectoris	FDA-approved drug (3244)	−6.55*	36
ALA-Protoporphyrin IX	actinic keratoses, photodynamic therapy	-	-	37
Ospemifene	breast cancer, postmenopausal symptoms	Selective estrogen receptor Modulators (SERMs)	−22.40**	38
Cetirizine	allergic rhinitis, dermatitis, and urticaria	FDA-approved drug (5883)	−7.60*	39
Nintedanib	idiopathic pulmonary fibrosis	Purchasable FDA-approved drug (1335)	−7.91*	40

For benchmark, the docking score of nutlin-3a is −7.7 kcal/mol (*Autodock Vina) & −32.38 kcal/mol (**Discovery studio)

S2. Origins of Redundant-Entry Bias

Table S2. Example of problem (i): Multiple IC_{50} records of nutlins-3a due to assay variations. *Which one should we keep in our dataset?*

Assay Type	pIC_{50}
ELISA (1)	7.21
ELISA (2)	6.22
HTRF	7.75
TR-FRET (1)	7.73
TR-FRET (2)	8.00
Cell line antiproliferative activity	5.82
fluorescence anisotropy competition	6.00
Fluorescence-polarization peptide displacement (1)	6.21
Fluorescence-polarization peptide displacement (2)	7.16
Surface plasmon resonance	7.05

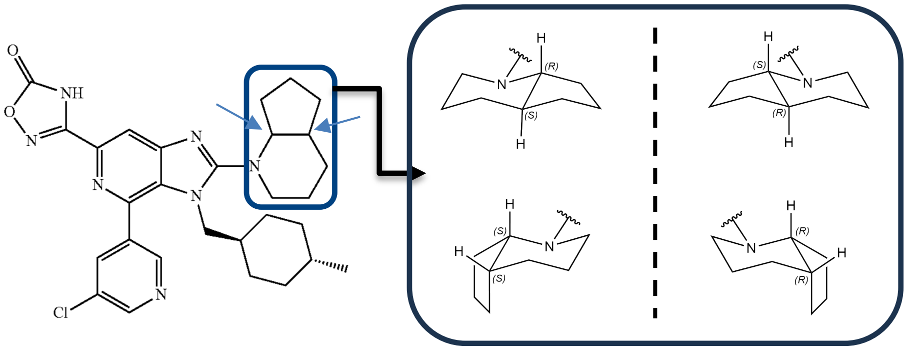

Figure S2. Example of problem (ii): Four stereoisomers corresponding to distinguished bioactivity values (IC_{50}: 63, 1, 2, 58 nM) had not been explicitly identified or specified; There was no known mechanism to assign which value belongs to which stereoisomers.

S3. Hit Optimization Setup

S3.1 Molecular Re-Docking Setup

For MOE docking, the target was selected atoms present around the binding site with an induced fit approach. The selection of the binding site was assisted by the MOE SiteFinder feature, which pointed out two well-defined pockets: Pocket-1 (p1) and Pocket-2 (p2), illustrated below. p1 is the p53-binding site, which serves as the primary pocket, whereas p2 is located between the two identical domains of MDM2. On the other hand, Autodock Vina uses a grid box to define the simulation area for docking 25 × 35 × 30 Å. In GOLD, we define the simulation site by determining the center point in the binding site and the distance to the center point, creating a spherical area.

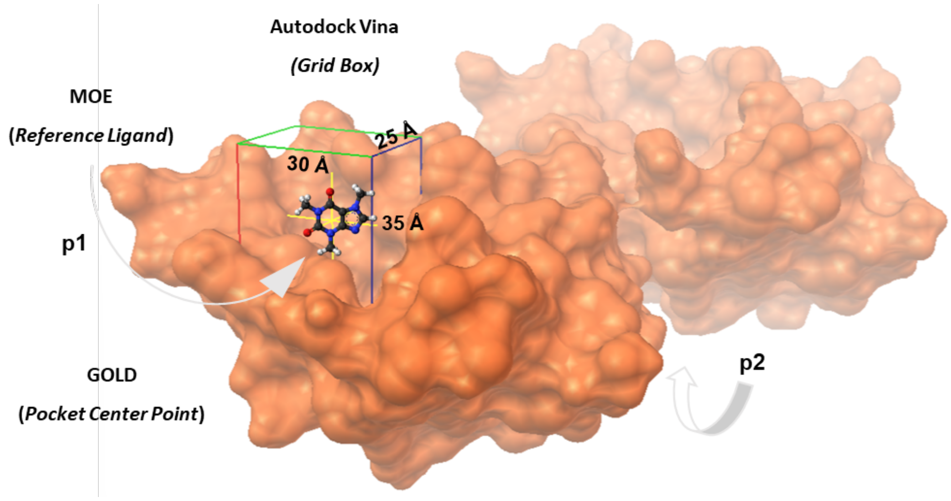

S3.2 Toxicity Prediction

Table S3. Multiple Toxicity Endpoint Predictions using ProTox-3.0 on H1 compounds

Ligand	Toxicity Class*	Toxicity Endpoints Activity (Probability)				
		LD_{50} (mg/kg)	Hepatotoxicity	Cardiotoxicity	Carcinogenicity	Mutagenicity
MP	4	1500	Active (0.51)	Inactive (0.76)	Inactive (0.62)	Inactive (0.62)
OT	3	160	Inactive (0.70)	Inactive (0.87)	Inactive (0.56)	Inactive (0.60)
AT	5	5000	Active (0.76)	Inactive (0.66)	Inactive (0.64)	Inactive (0.91)
BI	5	3200	Active (0.56)	Inactive (0.78)	Inactive (0.58)	Inactive (0.72)
DR	4	700	Inactive (0.66)	Inactive (0.74)	Inactive (0.57)	Inactive (0.64)

*Class I: fatal if swallowed ($LD_{50} \leqslant 5$)
Class II: fatal if swallowed ($5 < LD_{50} \leqslant 50$)
Class III: toxic if swallowed ($50 < LD_{50} \leqslant 300$)
Class IV: harmful if swallowed ($300 < LD_{50} \leqslant 2000$)
Class V: may be harmful if swallowed ($2000 < LD_{50} \leqslant 5000$)
Class VI: non-toxic ($LD_{50} > 5000$)

Active with High Probability 1 2 3 4 5 6 *Inactive with High Probability*

Probability Level

S4. Optimizing ML Model

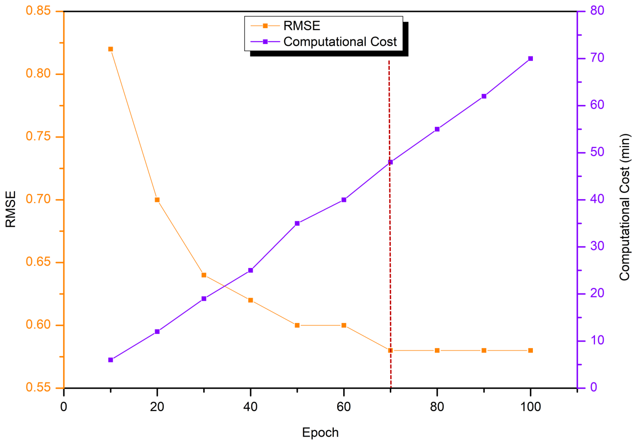

(a)

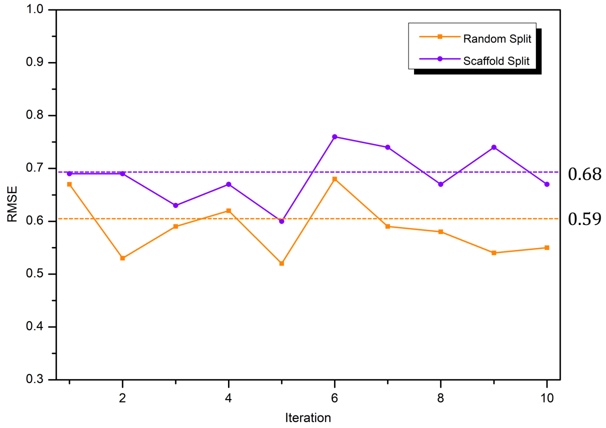

(b)

Figure S4. Performance evaluation of the predictive model throughout different steps in the optimization endeavors: (a) hyperparameter adjustment of epoch value and (b) generalization test running on 10-fold cross-validation

S5. Non-covalent Interaction Energetics

S5.1 MDM2-H2 Compounds Interaction

Table S5. NCI energy from MDM2-H2 compounds' complexes

Contact Residue	E_{EHT} (kcal/mol)	Frequency	Contact Area (Å²)	NCI other than vDW forces
MDM2-AT				
Arg65	−7.28	10	36.81	H-bond: N-H···O
Leu54	−6.46	28	56.1	H-bond: N-H···O
Phe55	−4.45	17	73.09	C-H···π interaction
Ile61	−3.66	12	26.69	-
Tyr67	−2.96	19	35.03	-
Ile99	−2.55	5	18.47	-
Phe91	−2.17	9	22.87	C-H···π interaction
Met62	−2.13	12	35.15	C-H···O interaction
Gly58	−1.91	30	29.55	C-H···O interaction
Phe86	−1.86	4	13.82	-
Gln72	−1.45	10	43.46	H-bond: O-H···O
Ile103	−0.92	3	8.07	C-H···π interaction
Val93	−0.8	7	28.11	-
Leu57	−0.68	4	13.69	-
MDM2-OT				
Lys51	−8.94	11	53.1	H-bond: N-H···O

Leu54	−4.54	21	58.25	C-H···π interaction
Pro20	−2.42	7	30.39	C-H···O interaction
Gln24	−2.2	9	17.39	-
Ile61	−1.86	5	21.99	-
His96	−1.74	7	16.41	C-H···π interaction
Ile99	−1.55	4	12.22	-
Val93	−1.03	12	46.33	C-H···O interaction
Phe91	−1.01	2	10.5	-
Met62	−0.42	4	17.54	
Leu57	−0.2	1	0.31	XB: Cl···O interaction
Phe55	−0.21	4	6.24	XB: Cl···O interaction

MDM2-MP

Leu54	−5.87	29	38.67	C-H--O interaction; C-H···N interaction
Ile61	−4.26	17	30.6	C-H--F interaction
Ile99	−3.81	13	33.51	C-H···π interaction; C-H···O interaction
Phe91	−3.47	20	21.17	-
His96	−2.02	8	24.54	C-H···π interaction; C-H···N interaction
Val93	−1.79	26	59.4	C-H···O interaction
Phe86	−1.53	10	11.67	C-H···F interaction
Val75	−1.13	2	8.81	-
Lys51	−0.55	25	86.61	C-H···π interaction
Met102	−0.33	1	5.46	-
Gln72	−0.31	3	8.44	-
Leu82	−0.24	3	3.3	C-H···F interaction
Ile103	−0.22	2	4.74	C-H···F interaction
Ser92	−0.02	2	2.36	-
Lys94	−0.01	1	0.21	-

S5.2 MDM2-p53 interaction

Significant Interaction with Helix-A

Binding Residue	Type of Binding	Binding Energy (kcal/mol)
Leu54	H-bond; CH···π interaction	-2.93
Met62	OH···S bond; CH···π interaction	-3.50

Significant Interaction with Loop-1

Binding Residue	Type of Binding	Binding Energy (kcal/mol)
Tyr67	π-stacking	-0.42
Gln72	CH···O bond	-4.45
His73	OH···π interaction	-0.44

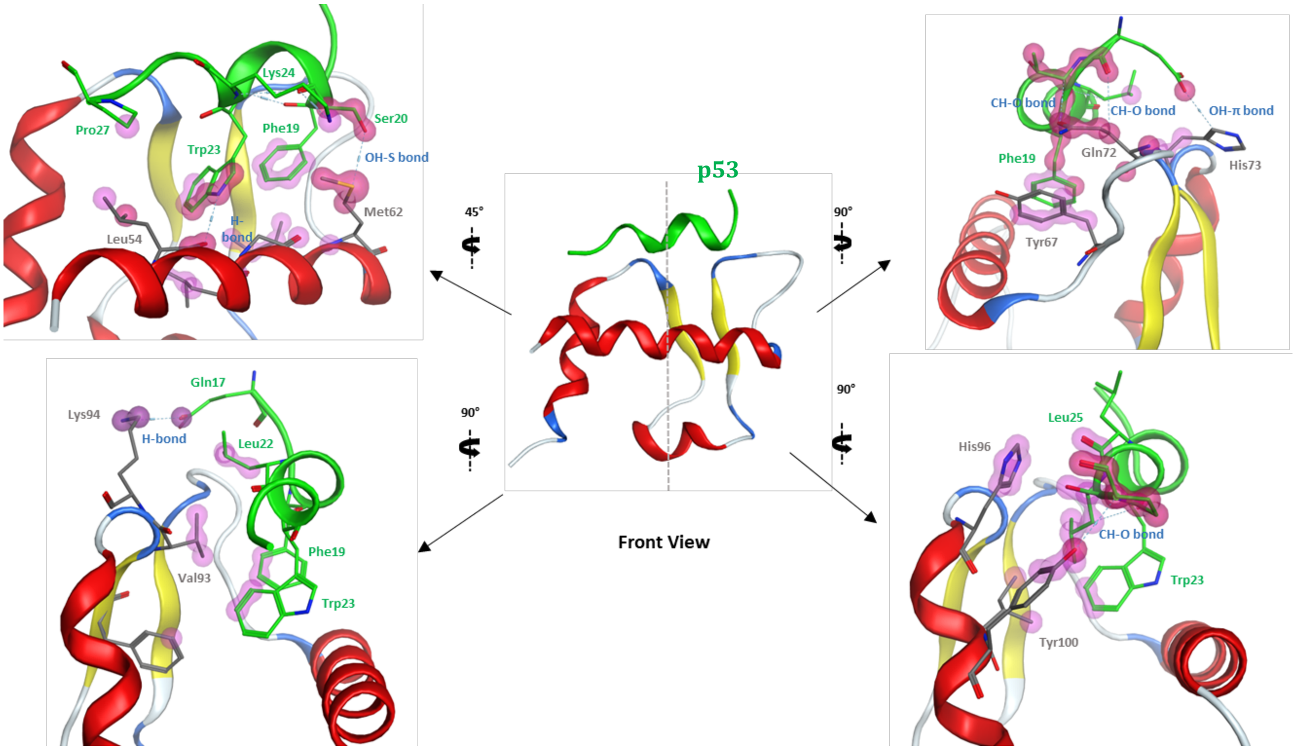

Significant Interaction with β-Sheet & Loop-4

Binding Residue	Type of Binding	Binding Energy (kcal/mol)
Val93	CH···π interaction	-0.78
Lys94	H-bond	-3.27

Significant Interaction with Helix-C

Binding Residue	Type of Binding	Binding Energy (kcal/mol)
His96	Weak H-bond	-0.78
Tyr100	CH···O bond	-3.27

S5.3 MDM2-NV Interaction

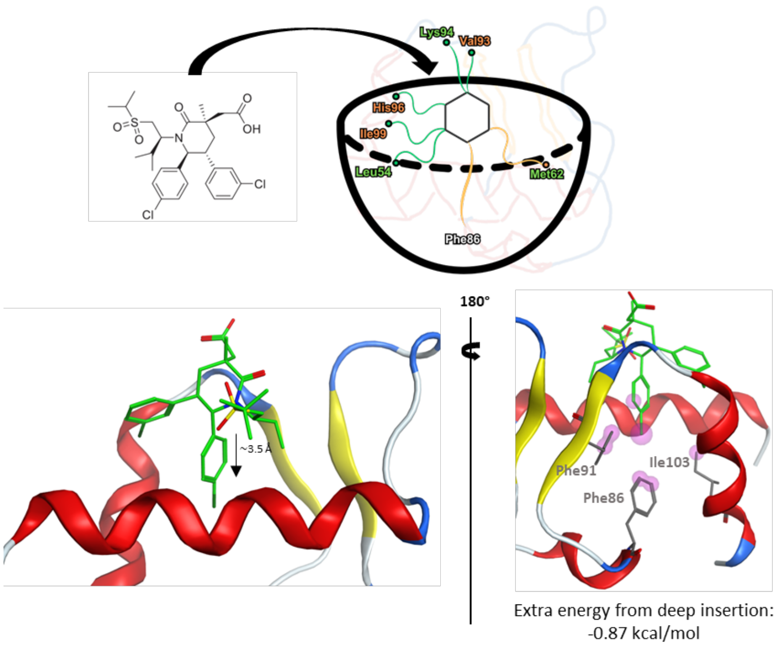

Extra energy from deep insertion: -0.87 kcal/mol

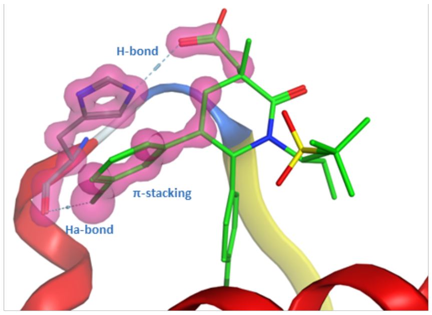

Location	Binding Residue	Type of Binding	Binding Energy (kcal/mol)
Helix-C	His96	Ha-bond; H-bond; π-stacking	-4.08
Loop-4	Lys94	H-bond	-3.64
Helix-C	Ile99	CH···Cl bond	-1.44
Helix-A	Leu54	CH···π interaction	-1.36
Loop-4	Val93	H-bond	-1.16
Helix-A	Met62	CH···S bond	-0.80
